# Phthisis Pulmonalis

**Published:** 1884-06

**Authors:** H. V. M. Miller

**Affiliations:** Professor Practice Medicine in the Atlanta Medical College, Atlanta, Ga.


THE ATLANTA

MEDICAL AND SURGICAL JOURNAL.

VOL. I. JUNE, 1884. No. 4.

### Original Communications.

### PHTHISIS PULMONALIS.*

BY H. V. M. MILLER, M. D.,

Professor Practice Medicine in the Atlanta Medical College, Atlanta, Ga.

From the earliest dawn of recorded time, Phthisis Pulmonalis has been known amongst mankind. For three thousand years the records of medicine have been burdened with horribly accurate pictures of the long series of unchanging symptoms which herald its armies of victims annually to the tomb.

Nor do these come from the ranks of infancy, from the aged or the debilitated, but nearly all of them from within "the conscript age," the period of youth or vigorous manhood or womanhood, and are cut down far short of three-score and ten years, the *ultima thule* of human hope and professional ambition. The thought excites sympathy, that it seems to select for its victims the fairest forms, the most engaging dispositions and the brightest intellects; its fatal shafts are directed with most mortal aim at the young, the beautiful and the most highly gifted.

Whether it is increasing or diminishing throughout the world is not now certainly known, but from the best statistics attainable, it may be safely estimated that at least one fourth of all the mortality of adults occurring amongst civilized people, is caused by consumption. In the United States alone not less than one hundred thousand people annually die of it. Not an hour strikes upon the horologue of time in which there are not, in our own country, half a score of victims stricken by this insatiate archer. Among all na-

* Read before the Medical Association of Georgia April, 1884.

tions, what is its destructive limit? The fatigued imagination shrinks aghast at the fearful aggregate.

What subject can more fitly engage the attention of a medical association than the consideration of any means of lessening the devastation of so dreadful a scourge? The history of professional effort in this direction continued for many centuries, is intensely interesting, but without reviewing this for the purposes of this paper, it will be necessary only to state in general terms some of the conclusions at which perhaps it may be said a majority of the profession have arrived:

1. The delusive hopes of enthusiasts; who have imagined that there have been, or may be, discovered antidotes or specifics or modes of treatment by drugs, curative of consumption, have vanished before the sad realities of impartial observation.

2. The opinion, at one time generally credited, that the disease consisted in the growth of a neoplasm, as malignant as cancer, as intractable in its progress and as fatal in its results, has been dissipated by the revelations of pathological anatomy, which have demonstrated that it has been in many instances, absolutely cured, and in a much larger number life indefinitely prolonged.

This belief, that consumption was inevitably fatal, has exercised a most malign influence over its treatment; it discouraged thought, stayed investigation, paralyzed effort, and medication dwindled into blind impotent routine, or turned recklessly to the dangerous experiments of unreasoning eclecticism.

Happily this philosophy of despair did not absolutely suspend professional exertion; something could be done to diminish evils confessedly irremediable, much to make comfortable or perchance prolong a life cheered and enlivened by a never-sinking hope of ultimate recovery; to this end medication in consumption has been mainly directed, satisfied that from the necessities of the case it could have no higher object or greater extent. Even within this limit its value has been incalculable; every step in the onward march of the malady has been watched with ceaseless vigilance. Every symptom has its appropriate palliative, every pain its anodyne, every imaginable suffering its placebo at least; the ever varying approaches of the attack are met by the almost infinite, though weaker resources of the defense. It is impossible to regard, without admiration, the ingenuity, the perseverance, the constant courage with which medical men have conducted this unequal war with symptoms. Every day, for ages, they have renewed the conflict, and every day have been vanquished. All must regret that the battle has not been fought upon a larger plane, with better weapons and at an earlier period.

3. An unprejudiced review of all the facts of its history appears to justify the conclusion that the starting point of consumption lies far back of the obvious symptoms which mark its progress, and consists in a perversion of the functions of innervation and nutrition, resulting sooner or later in such specific deterioration of the blood as renders it more or less unfit for its ordinary physiological functions. This deterioration may be favored by hereditary taint or predisposition and developed by bad air, bad food, bad digestion, or any other cause which diminishes the tone and vital power of the subject. The change is at first inappreciable, and by any known means of investigation undetectable, but sooner or later it manifests itself in the altered behavior of the plasma, exuding from the nutrient vessels of the tissues In healthy nutrition this exudation always occurring is quickly appropriated or absorbed and returned to the general current of the circulation. When in excess as in inflammation it first coagulates then softens and is absorbed or becomes organized into new tissue, or undergoes a lower form of organization into pus cells to be discharged from the body. The exuded plasma, by the influence of tubercular diathesis, is so lowered in its vitality as to be incapable of organization, even in its lowest form, it simply coagulates, is unfit for all nutrition and tends only to disintegration; these lifeless, heterologous coagulæ, large or small, singly or in battalions, are called tubercles.

From this point the history of consumption is the chronicle of the changes which occur in these chronic exudations, and of the irritation and pathological action which they establish in the tissues where they are deposited. The progress of these indicated by well known signs, it is unnecessary to follow. "Behold are they not written in the ten thousand forgotten volumes of professional lore?"

Accepting the pathological conclusions above stated, it follows that as Phthisis is a disease whose occurrence and malignancy are largely due to predisposition or initiatory blood change, the first indication is to remove or prevent this dyscrasia, whether it comes from inherited tendency or from accidental causes. The existence of the predisposition may be inferred with sufficient certainty from the general appearance and conformation of the subject, from the history of his ancestry, from his personal habits and mode of life, and from the general condition of his environment. This is the hopeful period for treatment, it is not safe to wait until the enemy is within the walls, it is dangerous to permit his entrance. Reasonable expectation of perfectly successful results can only be predicated upon wise prophylactic management, begun early and pursued with tireless painstaking constancy.

The obvious indications in all cases are to prevent or limit the tubercular deposit, to favor its removal and to fortify the system against the exhaustion attendant upon the pathological phenomena it excites.

The most important and approved means of filling them are, suitable nourishment, perfect assimilation, full respiration of pure, dry tonic atmosphere, at all times and everywhere, exercise proportioned to the vigor of the subject, the normal action of all the emunctories of the body, especially of the skin, and the selection of a suitable climate. The value of each of these has had the sanction of professional approbation in all ages, the combination of all of them is necessary to fully meet the emergency.

By suitable nourishment is not meant an abstemious or antiphlogistic regimen; influenced by erroneous pathological opinions, this has been fully tested and its bad results demonstrated. The diet in all cases of consumption should be generous; consisting of milk, cream, eggs, butter, bread, all kinds of animal and farinaceous food. Generous wine or a little stronger stimulants may be sparingly indulged in at dinner. As much variety as possible, good cooking and the best of viands should daily tempt the waning appetite. It is seldom necessary to limit the amount of food, the difficulty is to take enough, especially of the kinds containing oil; deficiency of this element is a fruitful source of evil; the children of the rich reject it, the offspring of the poor often cannot get it, and crowds of victims with excessively albuminous blood and consequent tubercular deposits are furnished from both of these classes. Judicious medical advice is never more serviceable than in carefully watching the effects of diet on each individual case and directing its regulation.

It must be remembered that the mere distention of the stomach, eating without the solicitation of appetite, may do more harm than good; digestion and assimilation must wait on appetite; the best stimulant to both is appropriate exercise, which by accelerating the circulation and respiration and producing natural waste of all the tissues of the body, excites hunger, which is nature's signal flag for distress, calling for new material to repair waste. It is useless to give nutriment even under the favorable conditions of pure air and a good climate, unless by exercise the air be forced into the lungs in increased quantity and circulated by means of the blood throughout the system.

Various methods of taking exercise have been suggested and the good effects of each recognized. Where all are confessedly valuable, convenience and adaptability will determine the best. Sydenham, *clarem et venerabile nomen*, declared that horseback riding was as perfect a specific in consumption as bark in ague; any movements which call into action the muscles attached to the chest are thought by many to be especially valuable, passive motion, as in carriages or otherwise, best suits the weak and debilitated, walking practicable to some extent in all weather will agree with the stronger. Slowly climbing a hill or mountain brings all the muscles into action and is a potent, but easily regulated, stimulant to both the respiratory and circulatory systems Violent and exhausting efforts should be avoided; it is better to take a little exercise at a time, but frequently every day, and to continue it regularly and methodically, increasing its amount and varying its character as the strength improves.

The scriptures declare that God breathed into his nostrils the breath of life and man became a living soul. Respiration is the essential function of vitality. The atmosphere and the lungs are its instruments, and its perfection demands the absolute integrity of both. Slight changes may be tolerated for a short period, but any continued deterioration of the atmosphere arrests most important functions, impairs health and endangers life. Pure air should contain no excess of carbonic acid, or any other gas not proper to its constitution, no excess of humidity, no vegetable or animal effluvia, no malaria, no floating particles of dust or matter of any kind, and none of the recently expired air of men or animals. Such air alone is fully fitted to the purposes of respiration; and a free supply of it at all hours of the day and night, summer and winter, is the *sine qua non* of all hopeful treatment of consumption. All considerations of convenience or expense should yield to this inexorable demand.

The breathing should be habitually deep and full. Diminished breathing space, congenital or acquired, is one of the earliest and most common signs of danger and a measure of the progress of disease. Every air cell should be properly filled with pure atmosphere; in this way alone aeration of the blood in the minute ramifications of the pulmonary artery is secured and its free circulation maintained. Loss or diminution of breathing capacity occurs most frequently at the apex of the lungs, the portion of them for anatomical reasons most difficult to inflate, in which locality nearly always tubercular deposit begins. Congenital narrowness of the upper part of the chest is common, and its danger admitted; in such subjects as well as in the debilitated, some attention and effort are required to expand the air cells at the apex of the lungs; the voluntary respiratory muscles must be called into action to supplement the involuntary; these from feebleness or partial atrophy do not readily and continuously respond, and the local retardation of the blood movement and increased exudation determines the preference of this locality for tubercular deposition. Soon the hollow chest, the drooping shoulder, the slender neck, the prominent clavicle, the projecting scapulæ and the dull resonance on percussion beneath the clavicle, announce the existence and indicate the cause of deposits.

This most alarming condition, like that of the innocent stag, that from the hunter's aim had taken a hurt, always excites sympathy, but it demands also prompt, careful and prolonged medical supervision. The greater the diminution of the breathing space, the purer should be the respired air; the greater the loss of muscular and vital tone, the more assiduous the efforts to restore it.

Numerous observations have demonstrated that contraction of the chest may be prevented, arrested, and in youth, even restored by judicious counteraction patiently continued.

All employments requiring a stooping, constrained posture should be promptly abandoned, the student must leave his books, the artisan his bench, the clerk his desk, and seek in other avocations the means and possibilities of continued life. By prolonged manly exercise the enfeebled respiratory muscles may regain their strength, the drooping carriage and tottering walk of the invalid may be exchanged for the erect attitude and buoyant step of the soldier, and the narrowing chest may in time recover its full expansion.

The interdependence of all the functions of the body is well known; the physiological action of one aids or supplements that of another and the disorder of one often reacts upon others; in the purely analeptic treatment of consumption this fact should be constantly remembered and every derangement carefully watched and promptly corrected.

The close sympathy between the lungs and the skin points to the latter as of special importance. Strong impressions made upon it by sudden alternations of temperature should be guarded against by suitable clothing. Unnecessary wrapping and swaddling, at all times debilitating, are hurtful; these, together with hot rooms, air-tight bed chambers and the artificial atmosphere created by heated stoves without proper ventilation, render abortive the most skillful management. The patient must be brought by training and habit to support any temperature, and in it to maintain the proper action of the skin. The atmosphere itself, with varying temperature, is the proper medium in which to begin the training, followed afterwards by daily bathing in water in the manner best suited to the power of reaction of the patient; perhaps with tepid water at first and the temperature gradually reduced even to the freezing point. Partial sponging may serve for a beginning, changed afterward to the sitz, plunge or shower bath.

The general tonic and stimulating effect of water thus used has been long known and highly commended; but besides this it promotes the exhalent and secretory offices of the skin, relieves and supplements the functions of the lung, accustoms the body to a lower temperature, and greatly lessens the susceptibility to atmospheric changes.

It will be perceived that each of the means here spoken of is only a part of one harmonious whole; little good will be derived from any one of them employed singly; they all work together and mutually aid each other in the production of common results, namely: to restore and stimulate all the complicated processes of sanguification, to augment appetite, to increase strength, to arrest the destructive progress of disease, and to begin and maintain recuperating physiological changes which shall terminate in restored health.

The effect of climate upon the development and progress of consumption has been long the subject of earnest study and investigation. Under the influence of varying pathological theories and different areas of observation, it is not surprising that conclusions are not as yet uniform or satisfactory. Phthisis pulmonalis may be met with in all zones of the earth's circumference, but there are places in each of them where it is comparatively rare. This comparative exemption is fairly attributed to local causes or climatic influence, and gives the highest prominence to the question of the selection of a place of residence, temporary or permanent, which will afford the greatest probability of continued health and long life.

In determining this question it may be assumed that the locality in which the patient sickens, the place in which his ancestors have died, the factory in which his comrades met their fate, the occupation and habits which he and they have followed are the worst for him, mere change to any other is often beneficial and in some instances effectual.

When liberty of choice may be exercised common sense would dictate the selection of the place in which the disease is most infrequent among the resident population. The safest place in a battle field is where the fewest soldiers are hit by the enemy's missiles. Theoretical opinions of what ought to be, or what, in view of the popular prejudices, probably will be, cannot settle a question so important. It must be left to the arbitrament of statistics.

While it is conceded that in certain places the inhabitants enjoy comparative immunity from consumption, it is lamentable that there are so few statistics proving indisputably the fact and indicating the degree and areas of immunity.

The opinions of local physicians not based upon tabulated cases are worth but little, and there has been no concerted effort to collect facts upon a scale broad enough to be perfectly satisfactory. Enough, however, is known to demonstrate that a cold climate does not produce it, a warm climate does not exempt from it. The most perfect reported immunity from it is in the Arctic region. It is extremely common within the tropics. Common consent, in the absence of reliable data, assigns the first rank in the scale of exempted areas on the American continent to the table lands of South and Central America; of Mexico, old and new, and the elevated plains bordering the Rocky Mountains in the United States. Without questioning the correctness of this assumption, it will be sufficient to say that these places are so remote and inaccessible as to deprive it of practical value.

The census of the United States, though very defective, gives upon this subject the only information approaching authenticity now attainable, and furnishes grounds for conclusions at least approximately true. In newly occupied territories the greater health and vigor of the first settlers, their habitual activity and free exposure to pure atmosphere at all times explains, perhaps, the temporary immunity from phthisis in them which the census shows to exist during the first few years of their occupancy. With this exception it is shown that the annual deaths from consumption vary in different States from one in three thousand to one in three hundred of the total population. The vast extent and diversities of climate in many of the States, forbid any satisfactory comparison on that basis.

In a general way it is proven that throughout all the Gulf and Atlantic States. all the Lake region, and the entire valley of the Mississippi and its tributaries as far west as the table land of the Rocky Mountains, the disease is fearfully prevalent, though in different degrees. The difference becomes more striking if the comparison is made by small districts or counties. By this test it is discovered that there are localities favored by very great, if not perfect, exemption from this disease; careful professional inquiry should ascertain and define their limits. Parallels of latitude do not bound them; mortality from consumption is as great in some sections of the South as in certain localities of the North. Statistics show that New York is not worse than New Orleans.

Careful examination of the census returns by counties proves the singular infrequency of the disease in a few localities in several of the States Without entering into detail and presenting an array of figures which would be tedious, it may be stated that, tried by this test, the best of all places "*facilis princeps*," are the elevated regions of Georgia, South and North Carolina, New Mexico and Colorado, in the order named.

It would be easy to argue that this revelation of the census ought to be true, since in every place in which immunity is indicated by it the conditions are present which are recognized by professional investigation as essential in preventing and curing this disease; but a point has been reached in this inquiry where demonstration, not argument, is demanded.

This can only be obtained by experiments broadly and intelligently conducted. At the most favorable points within the limited area of comparative immunity, as ascertained by observation and the census, there should be and will be as soon as professional conviction shall sanction it, resorts established and made pleasant, comfortable and attractive, and kept open during the entire year.

To these physicians could send their patients, in all stages of the disease, where, under the best medical supervision, a fair and impartial test could be made of the value of climatic treatment; where could be determined with accuracy the stages of the disease in which it is most beneficial, the particular seasons of the year and the length of residence necessary to secure it, and the particular class of cases if any, in which it is impotent or hurtful.

In the interior of some of the South Atlantic States there are localities, mostly dry, sandy ridges covered with pine forests, remote from the Atlantic and Gulf coasts, distant from rivers, lakes, marshes or swamps, of which Thomasville, in .Georgia, and Aiken, in South Carolina, may be cited as examples, where the native population rarely suffer from consumption. These places have been hopefully looked to as valuable sanitaria or safe places of residence. Doubtless there are conditions in which the mild, sedative atmosphere of these places may stay the progress or alleviate the symptoms of the disease, and it is desirable that the experiments now being made by invalids be continued until certainty is reached as to the proportion and kind of cases in which good results can be anticipated. That amendment here is universal, or even the rule, is unhapp'ly discredited by the facts.

One condition of the atmosphere present in all places most celebrated for the prevention or cure of consumption is lightness, the result of elevation. Under the influence of the now exploded error, that a cold climate produced consumption and a hot climate prevented it, large numbers of invalids every winter fled towards the tropics and the effects of altitude were overlooked. It is rare to find in old medical writings any reference to it; more recently it has challenged observation. Physicians sometimes advised a short mountain residence in summer to secure for their patients pure air and abundant exercise. Marked improvement of their condition excited surprise and astonishment, the advice was repeated until the phrase "the mountain cure" became proverbial. The number of phthisical patients visiting elevated places annually increased, and has more recently reached such proportions, and the cases have been so thoroughly studied, as to justify some conclusions in regard to it.

Without undervaluing the general influence on the constitution of pure air, abundant exercise and careful dieting, and apart from the asceptic, tonic influence of a mountain climate, confessedly valuable in all debilitating diseases, diminution of barometrical pressure, mere lightness of the atmosphere, slowly but surely induces certain changes in the thorax and physiology of respiration which are most potent helpers in the treatment of consumption.

In ascending from the sea level it is estimated that on the surface of an ordinary sized human body the atmospheric pressure is diminished at the rate of one thousand pounds for every thousand feet of elevation. Every dweller in low lands, on visiting high regions, is conscious of an increased feeling of elasticity, greater desire for exercise and greater ability to perform it, by reason of the greater rarity of the atmosphere, the breathing unconsciously becomes freer and fuller, and the expansion of the chest greater at each inspiration. The effect of this in more completely filling the air cells, especially in the partially closed and diseased portion of the lung at its apex, is easily understood, and the results upon the circulation and purification of the blood reasonably inferred.

In the United States physicians have usually been satisfied by noting the improved condition of patients after irregular sojourns in elevated places, but have not hitherto been able to study with sufficient care all the conditions necessary to form a decided opinion of its preventive or curative power. In other countries the inquiry has been further prosecuted, and apparently conclusive evidence obtained from various sources shows a marked and beneficial influence on the respiratory function.

At first the breathing rate is increased, but later on, as the lungs and thorax become expanded, the rate returns to the normal, but the inspirations are deeper and fuller. Increase of appetite generally appears early and shows itself in the large meals taken by persons of habitually capricious tastes. Gain in weight almost uniformly follows and increase of strength is exhibited in the greater ease with which the prescribed exercise is taken. The sweat glands recover tone and normal action, the night sweats gradually diminish and disappear.

The expansion of the thorax and accompanying modification of lung action is most remarkable. Its occurrence is not accidental but almost uniform, as often as ninety per cent. of carefully noted cases of phthisis in all stages of the disease. It is not conjectural but ascertained by actual measurement externally and by the spirometer.

The extent of chest expansion varies in different persons from one to three inches. It occurs in those who give no evidence of lung disease, but more markedly in those who do. The dilatation may be in all directions, but is most common in the antero-posterior; it is more frequent in the upper region of the chest, and the change in the shape as well as the size of the chest is very obvious and striking.

The length of residence required to produce these changes varies in different individuals, but presumably should extend over many months. The distending force is unquestionably the larger volume of rarefied air necessary to be inhaled at each inspiratory movement; it expands every ramification of the lung tissue as fully constantly as can be done temporarily by the aid of the voluntary respiratory muscles; this dilatation constantly maintained with every breath. finally restores the normal capacity. The lungs, the chest wall, all the tissues accommodate themselves to the altered condition, and in most patients the dilatation becomes permanent; in some, probably because of a return to lower places before the altered shape has become the fixed habit of the body, the contracted chest reappears. These changes of shape and size always indicated by corresponding alterations of the lung sounds on auscultation and percussion afford a means of measuring the progress to recovery.

If residence at high altitudes cannot be prolonged throughout the year, observation shows that a winter sojourn gives better results for the consumptive than one of like duration in summer. In debility from malaria or other causes the maxim is reversed.

The altitude to be preferred is not absolutely determined. Mountain peaks clothed with perpetual snow are uninhabitable; medium elevations, not too remote from the comforts of civilization essential to the invalid, are to be chosen. General concurrence of opinion among those who have given most thought to the subject indicate medium elevations of from two to four thousand feet above the sea level as the limits of greatest promise.

In the mountain regions of Georgia, South and North Carolina, as well as New Mexico and Colorado, all shown by the census tables to possess most extraordinary immunity from consumption, there are many localities having the climatic advantages of purity, salubrity, tonicity, medium humidity and temperature of the atmosphere, and at the same time sufficient elevation to thoroughly test the foregoing conclusions. Some of these are already well known to the seekers of pleasure, health, and leisure during the summer months, and the delightfully cool bracing air attracts them in annually increasing numbers; but none of them have been selected with special reference to effect upon tubercular disease. Further observation in regard to comparative humidity, of sudden changes of temperature, and exposure to currents of north winds, are desirable. But if the reasonable promise of ascertained facts be approximately fulfilled, the best places will soon be determined, where under the most favorable circumstances Hygeia and Therapia may unitedly triumph over a foe which has so often defeated them singly.

NOTE.—The highlands alluded to above include the counties of Gilmer, Fannin, Union, Towns, Lumpkin, White, Habersham and Rabun, in Georgia, portions of the upper counties of South Carolina, and nearly all of Western North Carolina.

The general height of this elevated plateau is from two thousand to three thousand feet above the sea level. From this plateau arise a vast number of mountain peaks whose summits reach from 3,000 to 6,780 feet of elevation.

Many of the villages and farm houses throughout all this region offer hospitality to sojourners during the summer months. Subjoined is a list of some of the most noted of them with the approximate altitude of each so far as ascertained:

| | |
|---|---|
| Waynesville, N. C | 2,756 feet |
| Asheville, N. C | 2,250 feet |
| Franklin, N. C | 2,141 feet |
| Webster, N. C | 2,200 feet |
| Highlands, N. C | 3,500 feet |
| Cæsar's Head, N. C | 3,200 feet |
| Mount Airy, Ga | 1,600 feet |
| Clarkesville, Ga | 1,500 feet |
| Tallulah Falls, Ga | 1,500 feet |
| Naucoochee, Ga | 1,400 feet |
| Dahlonega, Ga | 1,800 feet |
| Porter Springs, Ga | —— feet |
| Clayton, Ga | 2,000 feet |
| Rabun Gap, Ga | 2,200 feet |

